# Information flow to increase support for tidal energy development in remote islands of a developing country: agent-based simulation of information flow in Flores Timur Regency, Indonesia

**DOI:** 10.1186/s13705-021-00302-8

**Published:** 2021-07-21

**Authors:** Rohit Ramachandran, A. H. T. Shyam Kularathna, Hirotaka Matsuda, Ken Takagi

**Affiliations:** 1 Graduate Program in Sustainability Science-Global Leadership Initiative (GPSS-GLI), Graduate School of Frontier Sciences, The University of Tokyo, Room 334, Building of Environmental Studies, 5-1-5 Kashiwanoha, Kashiwa, Chiba 277-8563 Japan; 2 Department of Agricultural Innovation for Sustainable Society, Faculty of Agriculture, Tokyo University of Agriculture, Tokyo, Japan; 3 Department of Ocean Technology, Policy, and Environment, Graduate School of Frontier Sciences, The University of Tokyo, Kashiwa, Chiba Japan

**Keywords:** Tidal energy, Public support, Information flow, Social hierarchy, Multinomial probit, Agent-based simulation, Developing country, Indonesia

## Abstract

**Background:**

Public awareness is crucial for successful deployment of tidal energy, a renewable energy source that can provide clean electricity to remote islands. However, considering public attitudes on tidal energy are not well known, especially in developing countries, a barrier exists in implementing public engagement strategies. This study aims to contribute by identifying strategies for information provision—the initial step in public engagement—and estimate how these can be engaged to enhance support for tidal energy among the local public in a remote area of a developing country, in this case, Flores Timur Regency, Indonesia, considering their socio-cultural background.

**Methods:**

In this paper, we employ statistical analyses using multinomial probit modelling to identify the key variables that shape information flow. The aptness of the variables is then verified using post-estimation techniques for their use as input parameters for the simulation of the information flow in the field study area. Agent-based simulation (ABS) is employed to replicate the actual conditions in Flores Timur Regency, Indonesia, and simulate the flow of information through the local community.

**Results:**

According to the multinomial probit estimations, the people belonging to the top hierarchical group show a higher probability to support tidal energy compared to the members belonging to the lower groups. Understandably, around twice as many information flow cycles are needed to disseminate information to the members of the lowest hierarchical group, compared to the members of the top hierarchical group. The results also show that increasing the amount of available information has a positive impact on information dissemination.

**Conclusions:**

This study demonstrated that information provision is highly effective with propagation of information that specifically highlights the individual benefits, rather than the community benefits of tidal energy. Additionally, savings in terms of costs, time, and efforts can be realized if the most influential members of the local community are targeted initially before including all other stakeholders. The study also indicated that locals absorb more information and increase their support for tidal energy when additional data is made available. Finally, as long-term strategy, information provision becomes most effective when the local population gains higher educational capabilities.

**Supplementary Information:**

The online version contains supplementary material available at 10.1186/s13705-021-00302-8.

## Background

As widely recognized, fossil fuel usage for energy production is one of the main factors accelerating climate change, the most ominous threat globally [1]. Furthermore, use of renewable energy technologies contributes to climate change mitigation and disaster risk reduction, as highlighted in Goal 7 of the Sustainable Development Goals (SDGs) that promotes affordable and clean energy by all the member states of United Nations. According to the latest data about the progress towards SDG 7, stepped-up efforts towards all targets were urgently required if SDG 7 should be reached within the coming decade [2]. Tidal current energy is a new technology that can provide clean renewable energy and contribute to successfully attaining SDG 7. Tidal current energy can be considered superior to other renewable energy sources such as wave power or wind power, as it is less susceptible to severe weather events such as typhoons or storms and, compared to those sources, tidal currents are a regular and predictable source of energy [3, 4]. With several tidal projects under development in various parts of the world [5] there is an urgent need to address certain research areas. While there are many studies focusing on the technological [6–9], environmental [10, 11], and economic [10, 12–15] aspects of tidal energy, there are only a few studies dealing with the social aspects [16]. This is expected as tidal technology is relatively new and has not yet been implemented commercially leading to relatively scarce information about, especially among the general public.

Additional studies on social aspects are needed as large-scale renewable energy projects can only be successful with the understanding and support of the local public [17], highlighting the importance of public participation. Moreover, with many businesses involved in tidal energy development, the role of the private sector in public engagement remains unclear, emphasizing an urgent research need that needs to be addressed.

Most of the public participation strategies can be classified into three main categories: (i) information provision, (ii) consultation, and (iii) engagement [18]. Logically however, the last two strategies can be successful and efficient, only after the first strategy has been effectively implemented. Strategies focusing on consultation processes, whereby the public is formally allowed to voice their opinion, can only be effective if the public is sufficiently and correctly informed of all the related aspects of tidal energy, as it has been found that the public can form opinions inconsistent with prior scientific studies in case of offshore renewable energy [19]. Likewise, the engagement methods, when the public is involved in each stage of development, are bound to failure without adequately knowledgeable members. Providing information about benefits and disadvantages of the project at an early stage allows people time to think about the issues, consider implications, and formulate their views. An informed public will understand the trade-offs, be able to contribute meaningfully to project design, and have greater trust with the project proponent [20].

The importance of information provision is emphasized more if the awareness level of the local population is relatively low, for example in remote isolated islands in developing countries where favourable conditions for tidal energy development persist as revealed by Ramachandran et al. [21]. Therefore, when it is related to a new technology such as tidal energy, most aspects of which are largely unknown to the general public, information provision is not only the initial process for public engagement, but also the most vital.

For efficient information provision, the developers and policy makers need to ascertain how and when the information has to be disseminated for spreading it effectively throughout the community [22]. The developers not only have to identify the type of information to be diffused, but also the fastest and most efficient way considering the associated costs, time and effort [23]. Moreover, information dissemination should be tailored to the predilection of the intended audience, i.e. the local community. Hence, the study of the community, including its demography, its social structures, and local information preferences is paramount for the information dissemination process to be successful.

To provide a unique voice toward tidal energy development, this study focuses on the information flow for promotion of tidal energy in a remote isolated area of a developing country. This research adds on to the previous study by Ramachandran et al. [21] which estimated the support for tidal energy based on information provision, identified the key factors that impact information provision in the local community, and explored the impact of information provision for developing countries compared to advanced nations. This study also directly contributes to the agenda for social research in marine renewable energy [16] by focusing on the communication and knowledge flow in the community and supplements the previous study by Ramachandran et al. [21] by identifying the most efficient strategies for information provision.

This study uses multinomial probit regression model to estimate the probability of support for tidal energy in Flores Timur Regency as well as verify the aptness of the key variables identified in the previous study [21]. After the suitability of the variables is confirmed, the variables are used as input parameters for agent-based simulation (ABS). ABS, an ideal methodology to replicate social systems, is used in this study to simulate the flow of information in a remote local community in a developing country to increase support for tidal energy, reflecting the unique nature of their society, and the type of information that is preferable to them. The key factors affecting the information flow and the ideal strategies for information provision that can be used by the developers to increase support for tidal energy are explored in this study. The subsequent sections describe the study area, followed by explanation of the methods used for this study. The results are then described, before the discussion of key findings, and the study’s conclusions and limitations.

## Study area

The limited studies focusing on the socio-economic or developmental aspects of tidal energy are based mostly in advanced countries, highlighting a need for case studies from developing areas. There are many areas in developing countries such as Indonesia, where there is tremendous potential for tidal energy [24, 25]. Moreover, many areas with potential for tidal energy generation lie in remote locations, such as isolated islands. Larantuka strait in Flores Timur, a far-flung regency in Nusa Tenggarah Timur province in south-east Indonesia is one of the most favourable locations for tidal energy generation. Larantuka Strait is about 8 km in length. The north and south inlets of Larantuka Strait are both around 4.5 km wide. Meanwhile, at the narrowest part, the width of the strait is approximately 600 m and the depth is 20 m [26]. These conditions provide Larantuka strait with high potential for tidal energy generation [27–33]. While Larantuka strait is the potential site of the tidal energy project, entire Flores Timur Regency is selected as the study area, as its population will be the major direct beneficiary of the project. This study adds on to the previous study by Ramachandran et al. [21] examining the public acceptance of tidal energy in Flores Timur Regency.

The population in Flores Timur Regency is spread over 250 villages in the three habited islands of Flores, Adonara, and Solor, with the regency capital based in Larantuka in Flores. The social stratification in Flores Timur, with an active traditional structure, is highly prominent. The leaders of Koten, Kelen, Hurint and Maran clans maintain a revered place at the top of the hierarchy, followed by leaders of other clans and village elders [21]. Not only are the social structures made of at least four hierarchical levels, the governmental administrative bodies as well as the prominent religious institutions also display a hierarchical nature, with four or more stratifications. Based on interviews with the local community members and local government officials, it was identified that the social structure of the community is significant for all communications, as the information flow follows the top-down hierarchy [21]. This study considers the population of Flores Timur Regency, with its hierarchical society in replicating the flow of information, in relation to tidal energy development. The study also considers two types of information that may impact the public view on tidal energy. Statistical results of Ramachandran et al. [21] show that the local people in Flores Timur Regency tend to show more support for tidal energy if their preference is for information related to individual benefits of tidal energy, as opposed to the information about collective benefits of tidal energy.

## Methods

**Fig. 1:**
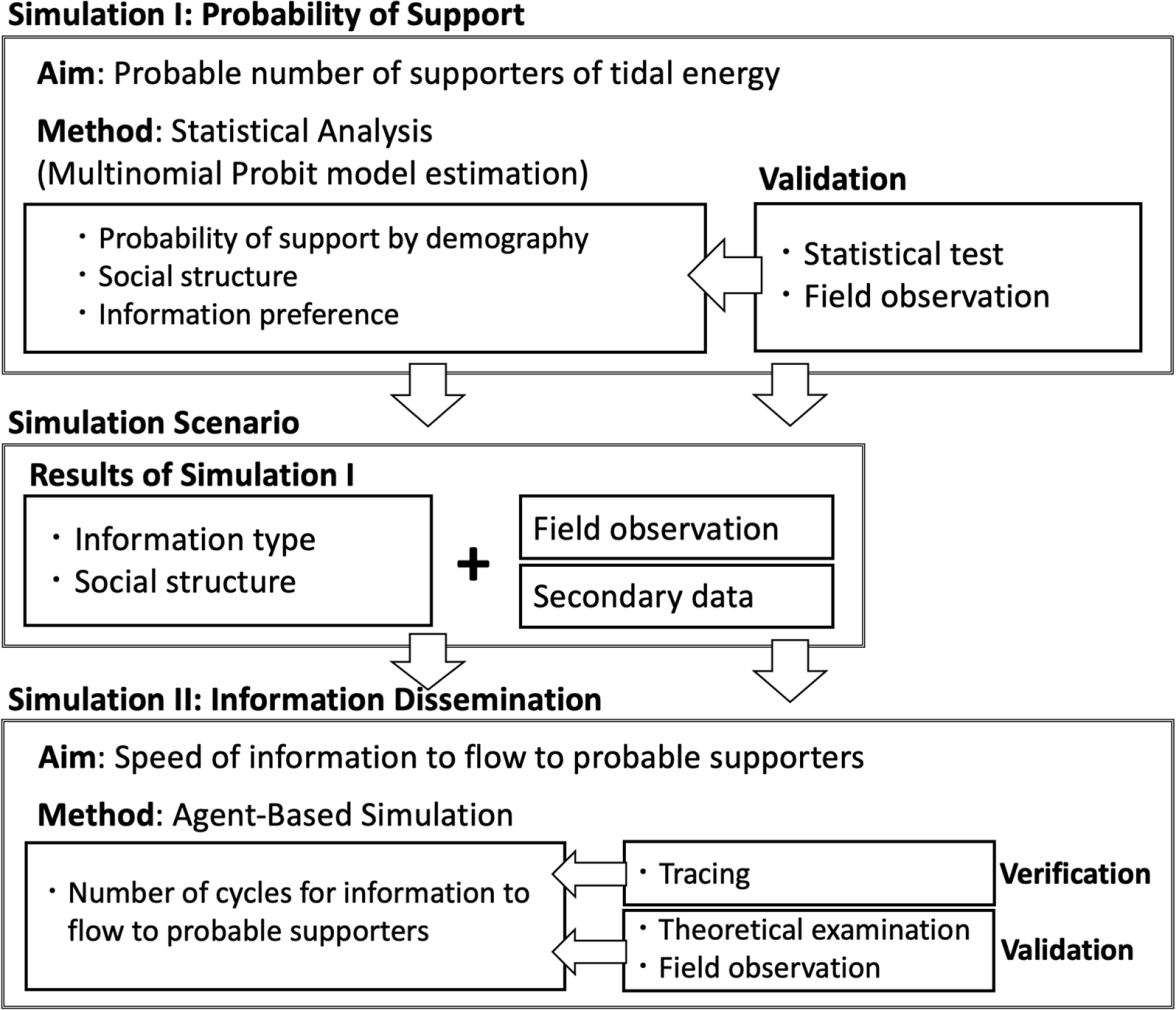
Framework for estimating support for tidal energy based on information provision

The support for tidal energy based on information provision is simulated according to the framework shown in Fig. 1.

Two kinds of simulation models are employed here: multinomial probit model and agent-based simulation (ABS) model. In the first simulation, multinomial probit regression modelling is used to estimate the probable number of supporters of tidal energy based on the information preferences, the demographical features, and the social structure of the local community. The output of the multinomial probit model is then used to create input parameters for the second simulation, the ABS model for information dissemination. Post-estimation techniques are then used to authenticate the suitability of the key variables to be used as input parameters for ABS, as has been employed in several studies [34]. As part of the post-estimation treatments, the variables are then distributed in hierarchical groups that represent the social structure of Flores Timur Regency, and the probability of each group to support tidal energy is estimated. Finally, the information flow in the local community is simulated using agent-based simulation (ABS) and the number of cycles required to provide information to the probable number of supporters of tidal energy is tallied for each hierarchical group based on the type of information.

### Step I: multinomial probit model estimation

The multinomial probit model, one of the most satisfactory choices for discrete choice modelling [35], is used to estimate the probability of support for tidal energy and is derived from the data and results presented in the previous article by Ramachandran et al. [21]. The data were collected from 135 respondents using questionnaires presented to residents, selected at random, from villages spread over the three major islands in Flores Timur Regency. The key variables as identified in the previous study, and used here for further analysis are described in Table 1.

**Table 1:** Description of variables

Variable	Description	Response scale
Collective outcome favourability	Respondent’s inclination towards supporting initiatives that focus on collective benefits of tidal energy	0—disagree 1—neither agree nor disagree 2—agree
Individual outcome favourability	Respondent’s inclination towards supporting initiatives that focus on individual benefits of tidal energy	0—disagree 1—neither agree nor disagree 2—agree
Education	Education level of respondent	1—no formal education 2—primary school 3—junior high school 4—senior high school 5—bachelors degree 6—masters degree
Age	Age of respondent	1—15 to 24 2—25 to 34 3—35 to 44 4—45 to 54 5—55 to 64 6—over 64

The multinomial probit model is then put through post-estimation techniques to verify the suitability of the variables for reflecting the actual situation in the case study area, and to be used as input parameters for the simulation.

The post-estimation techniques and the simulation processes are described below.

#### Post-estimation measures

After estimating the probability of support, two post-estimation measures are used to check the viability of the variables: goodness-of-fit and receiver operating characteristic (ROC) curves.

The key goodness-of-fit measure used here is Count *R*-squared. Count *R*-squared transforms the continuous predicted probabilities into a binary variable on the same scale as the outcome variable and then assesses the predictions as correct or incorrect [36]. The *R*-squared is this correct count divided by the total count; *R*-squared values greater than 0.5 suggest a good fit.

To further confirm the appropriateness of the variables, receiver operating characteristic (ROC) curves are also employed. In analysis for count data such as probit regression, ROC curves are very useful for evaluating the predictive accuracy of a chosen model [37]. For ROC curve, the predicted values generated by the probit model can be viewed as a continuous indicator to be compared to the observed response variable. ROC curves can be best interpreted by creating a graph using the variables to have a good visual representation of their accuracy. Additionally, a numerical measure of the accuracy can be obtained by calculating the area under the ROC curve, with the resulting values above 0.5 pointing to the accuracy of the variable.

#### Probability at specific values

The variables employed in the multinomial probit cannot be used as the input parameters for the ABS in the current form. The values for age and education variables in the multinomial probit model, ranging from one to six, can be directly used in ABS to indicate the demographic aspects. But the variables for individual outcome favourability and collective outcome favourability in same multinomial probit model, with values ranging from zero to two, need to be modified for use in ABS to correspond to the spreading of different types of information: information about individual benefits and information about collective benefits of tidal energy.

Normally, multinomial probit modelling uses the average response values of the variables of the entire sample to estimate the predicted probabilities. However, the social hierarchies consisting of four hierarchical groups that are observed in the society in Flores Timur have to be replicated within the ABS model with each hierarchical group having separate input values for each variable rather than the overall mean value of the entire sample. To account for the above conditions, multinomial probit is employed again to estimate the probability to support tidal energy when specific variable values are assigned to each of the four hierarchical groups as observed in Flores Timur Regency [21].

### Simulation scenario

As described in Fig. 1, scenarios are created using the results from multinomial probit model. Accordingly, three cases are created to reflect the wide range of values for the four variables: individual outcome favourability, collective outcome favourability, age, and education in the local society. Based on field observation of the hierarchical nature of the society and secondary data based on census that show the economic disparity between different classes, specific values then are assigned to the variables with the assumption that these values capture the specific social features to create an accurate representation of the local society of Flores Timur Regency. After specific values are assigned to the variables, multinomial probit is used to provide the predicted probability of each hierarchical group to support tidal energy, under the three cases. Once the support probabilities are estimated and the probable number of supporters under each case and hierarchical group is deduced, the next simulation process, agent-based simulation (ABS), is employed to simulate the number of cycles required for information to flow to the probable supporters of tidal energy.

### Step II: agent-based simulation (ABS)

Agent-based simulation (ABS) engages independent agents operating within a spatial grid that represents the agents’ environment. Each cell (patch) in the grid representing a geographical space where the agent resides, and each agent, run a particular set of rules and instructions that describe how they interact with other cells or agents. The macro-behaviour of the system may look different compared to the individual behaviour of the agents [38].

ABS is a good choice for the present research because the information sharing process being studied takes place between individual people (agents), and it is easy to see the spatial grid as representing the agent’s location within the community. Rules can be used to describe how stakeholders decide how to harvest or share information, and the outcomes of interactions between agents. Hence ABS can be seen an alternate way to replicate and describe social phenomenon as pointed out by Epstein and Axtell [39].

Moreover, ABS is an appropriate methodology to study occurrences that may transpire only in the future [40–44]. Additionally, with the study area being remote and isolated, ABS is an efficient and practical methodology to employ for conducting research. This study will use ABS to study the flow of information through the local community of Flores Timur Regency, respecting the structure of the community and the hierarchical rules of the society, for successful deployment of tidal energy.

### The ABS model for information flow

Information flow models have relied on certain specific aspects to investigate the spread of information, for example, the mode of information diffusion [45] or the role of influential stakeholders [46]. This model follows the approach similar to those employed by Wilensky [47] and Huang et al. [48] for their models. In this simulation model, the agents exist in a two-dimensional space, consisting of 2500 cells or patches, with an agent occupying each cell, with the proximity of two agents representing geographical distance. According to Allen [49], the physical proximity has an indirect relationship on the probability of agents communicating, and this is reflected in the model where agents who are close together are more likely to interact. The model uses spatial proximity, defined here as ‘visibility range’ to determine the vicinity where a given individual can seek information from or spread it to other agents. The results of the model simulations show the fastest and the most efficient way to spread information in the local community.

In ABS, the major simulation processes rely on the interaction between different agents, the interaction between the agent and the environment, and the rules guiding these interactions. The behaviours, interactions, and processes governing this ABS model are described below.

#### Agent behaviour

**Fig. 2:**
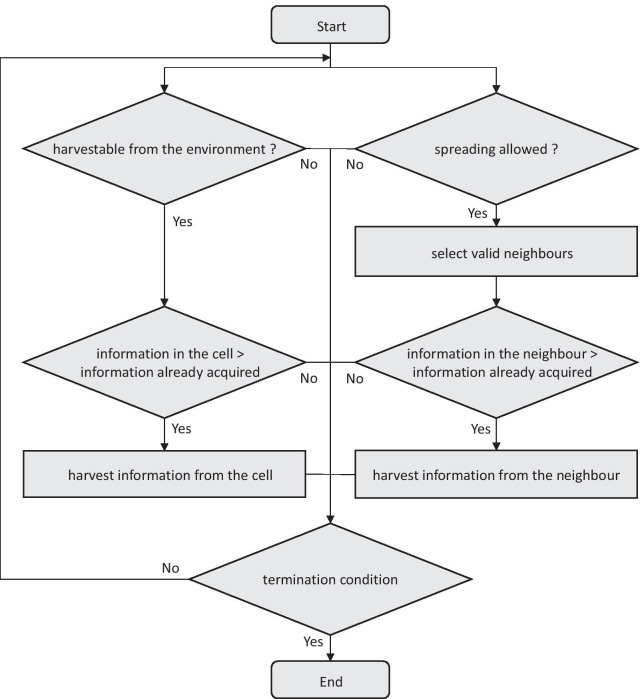
Flowchart showing the behaviour of the agents for one cycle

In our model, agents choose among two alternatives to absorb information: (i) From a valid neighbouring agent who has more information than itself or (ii) from its residing cell if information harvesting is allowed and the cell has more information than the agent itself. The behaviour of the agents as assumed in this study is shown in Fig. 2.

Information harvesting from the environment (residing cell) is defined according to Eqs. (1) and (2). The glossary of terms used to denote the harvesting and sharing procedures are presented in Table 2.

**Table 2:** Glossary of notations to describe harvesting and spreading of information

Harvesting from cell to agent	
${H_{C_{i,t}}}$	Harvested c-info amount by the agent *i* at time *t*
${{C_{i_{ i,t}}}^{\mathrm{cell}}}$	c-info in the cell of agent *i* at time *t*
${{C_{i_{ i,t}}}^{\mathrm{agent}}}$	c-info in the agent *i* at time *t*
${C_{i_{H,i}}}$	c-info harvesting rate of agent *i*
${H_{I_{ i,t}}}$	Harvested i-info amount by the agent *i* at time *t*
${{I_{i_{ i,t}}}^{\mathrm{cell}}}$	i-info in the cell of agent *i* at time *t*
${{I_{i_{ i,t}}}^{\mathrm{agent}}}$	i-info in the agent *i* at time *t*
${I_{i_{H,i}}}$	i-info harvesting rate of agent *i*
${A_i}$	Age of agent *i*
${E_i}$	Education level of agent *i*
Spreading from agent to agent	
${S_{C_{ i,j,t}}}$	c-info sharing from agent *i* to agent *j* at time *t*
${S_{I_{i,j,t}}}$	i-info sharing from agent *i* to agent *j* at time *t*
${C_{i_{R,j}}}$	c-info receiving rate of agent *j*
${I_{i_{R,j}}}$	i-info receiving rate of agent *j*
${F_i}$	Influence level of agent *i*
${S_{C_{i}}}$	c-info spreading rate of agent *i*
${S_{{I}_{i}}}$	c-info spreading rate of agent *i*
Spreading from cell to cell	
${E_{{C}_{ i,j,t}}}$	c-info spreading from cell *i* to cell *j* at time *t*
${E_{{I}_{ i,j,t}}}$	i-info spreading from cell *i* to cell *j* at time *t*
${{C}_{i_{{N}}}}$	Natural spreading rate of c-info
${{I}_{i_{{N}}}}$	Natural spreading rate of i-info

c-info denotes information about collective benefits of tidal energy

i-info denotes information about individual benefits of tidal energy

1 $$H_{{C_{{i,t}} }} = \left\{ {\begin{array}{*{20}l} {\left( {C_{{i_{{i,t}} }}^{{{\text{cell}}}} - C_{{i_{{i,t}} }}^{{{\text{agent}}}} } \right)*A_{i} *E_{i} *C_{{i_{{H,i}} }} /36;} & {C_{{i_{{i,t}} }}^{{{\text{cell}}}} > C_{{i_{{i,t}} }}^{{{\text{agent}}}} } \\ {0;} & {\text{Otherwise}} \\ \end{array} } \right.,$$

2 $$H_{{I_{{i,t}}}} = \left\{ {\begin{array}{*{20}l} {\left({I_{{i_{{i,t}} }}^{{{\text{cell}}}} - I_{{i_{{i,t}} }}^{{{\text{agent}}}} }\right)* A_{i}* E_{i}* I_{{i_{{H,i}} }}/36;} & {I_{{i_{{i,t}} }}^{{{\text{cell}}}} > I_{{i_{{i,t}} }}^{{{\text{agent}}}} } \\ {0;} & \text{Otherwise} \\ \end{array}}.\right.$$

At each time step or cycle, if harvesting is allowed, each agent’s information levels are updated according to Eqs. (3) and (4):

3 $${{C_{i_{ i, t+1}}}^{\mathrm{agent}}} = {{C_{i_{ i,t}}}^{\mathrm{agent}}}+ {H_{C_{i,t}}},$$

4 $${{I}_{i}}_{ i, t+1}^{\mathrm{agent}} = {{I}_{i}}_{ i,t}^{\mathrm{agent}} + {{H}_{I}}_{i,t}.$$

Information sharing among valid neighbouring agents is defined according to Eqs. (5) and (6). Selection of the neighbouring agent is directed according to the hierarchy level of the considered agent, level of information held and the visibility range.

5 $${S_{C_i,j,t}}=\left\{\begin{array}{*{20}l} {\left({{C_{i_{i,t}}}^{\text{agent}}}-{{C_{i_{j, t}}}^{\text{agent}}}\right)*{F_i}*{S_{C_i}}*{A_j}*{E_j}*{C_{i_{R,j}}} /36;} &{{C_{i_{i,t}}}^{\text{agent}}} > {{C_{i_{j,t}}}^{\text{agent}}} \\ {0;} & {\text{Otherwise}} \\ \end{array}\right.,$$

6 $${S_{I_{i,j,t}}}=\left\{\begin{array}{*{20}l} {\left({{I_{i_{ i,t}}}^{\mathrm{agent}}}- {{I_{i_{ j,t}}}^{\mathrm{agent}}}\right)*{F_i}*{S_{I_{i}}}*{A_j}*{E_j}*{I_{i_{R,j}}}/36;} & {{I_{i_{ i,t}}}^{\mathrm{agent}}}> {{I_{i_{ j,t}}}^{\mathrm{agent}}} \\ {0;} & \text{Otherwise} \\ \end{array}.\right.$$

At each time cycle, if spreading is allowed, each agent’s information levels are updated according to Eqs. (7) and (8):

7 $${{C_{i_{ j, t+1}}}^{\mathrm{agent}}} = {{C_{i_{ j,t}}}^{\mathrm{agent}}}+ {S_{C_{i,j,t,}}}$$

8 $${{I_{i_{ j, t+1}}}^{\mathrm{agent}}} = {{I_{i_{ j,t}}}^{\mathrm{agent}}} + {S_{I_{i,j,t}}}.$$

#### Cell behaviour

Similar to information sharing among agents, information spreading from one cell to another neighbouring cell materializes according to Eqs. (9) and (10):

9 $${E_{C_{ i,j,t}}}=\left\{\begin{array}{*{20}l}{\left({{C_{i_{ i,t}}}^{\mathrm{cell}}}-{{C_{i_{j, t}}}^{\mathrm{cell}}}\right)*{C_{i_N}};} & {{C_{i_{ i,t}}}^{\mathrm{cell}}}> {{C_{i_{j,t}}}^{\mathrm{cell}}} \\ {0 ;} & \text{Otherwise} \\ \end{array},\right.$$

10 $${E_{I_{ i,j,t}}}=\left\{\begin{array}{*{20}l}{\left({{I_{i_{i,t}}}^{\mathrm{cell}}}-{{I_{i_{j, t}}}^{\mathrm{cell}}}\right)*{I_{i_N}};} & {{I_{i_{ i,t}}}^{\mathrm{cell}}}> {{I_{i_{j,t}}}^{\mathrm{cell}}} \\ {0;}& {\text{Otherwise}} \\ \end{array}.\right.$$

At each time cycle, if cell level natural spreading is allowed in the environment, each agent’s information levels are updated according to Eqs. (11) and (12):

11 $${{C_{i_{ j, t+1}}}^{\mathrm{cell}}} = {{C_{i_{ j, t}}}^{\mathrm{cell}}}+ {E_{C_{i,j,t}}},$$

12 $${{I_{i_{j, t+1}}}^{\mathrm{cell}}} = {{I_{i_{ j, t}}}^{\mathrm{cell}}} + {E_{I_{i,j,t}}}.$$

Any random cell within the visibility range can be selected as the neighbouring cell for information sharing.

**Fig. 3:**
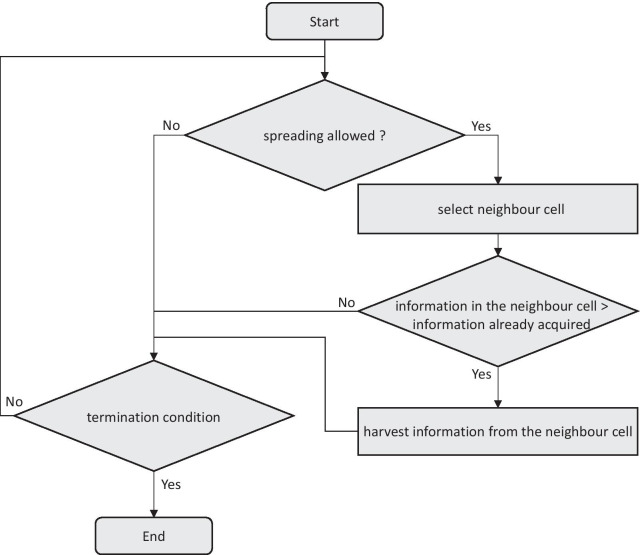
Flowchart showing information flow between cells for one cycle

The overall behaviour of the information flow from cell to cell is captured in Fig. 3.

#### The ABS model for information flow in Flores Timur Regency

In this model, the agents are classified into four groups reflecting the generic structures of Flores Timur Regency. As per the hierarchical nature of the local society observed in the field survey [21], information only flows to the agents in the adjacent hierarchy as shown in Table 3.

**Table 3:** Characteristics of agents from different hierarchies

Agent	Hierarchy			
	Level 1	Level 2	Level 3	Level 4
Stakeholder type	Elected officials, senior regency officials, traditional, clan leaders, senior priests	Senior district officials, heads of villages, Business owners, clergy	Junior village officials, traditional village elders, community leaders	Fishermen, farmers, employees, other local residents
Distribution of agents	6%	14%	32%	48%
Influence	Very high influence	High influence	Mid influence	Low influence
Info sharing With other hierarchical agents	Level 1 Level 2	Level 1 Level 2 Level 3	Level 2 Level 3 Level 4	Level 3 Level 4

Table 3 also highlights the conditions that govern the interaction between agents of different hierarchical groups. Level 1 agents represent the most educated and highly influential locals at the top of the hierarchical structure such as the regency mayor or bishop of the diocese, followed by level 2 agents consisting of high influence locals such as senior regency officers, senior priests, etc. Level 3 and level 4 agents are lower in the hierarchical structure consisting of local people such as fishermen and farmers. In this model, level 1 agents can only share information with other level 1 and level 2 agents, while level 2 agents can share with level 1, level 2, and level 3 agents. Likewise, level 3 agents can share with level 2, level 3, and level 4 agents, but level 4 agents can only share information with level 3 and level 4 agents.

In addition to the demographic factors—age, and education, this ABS uses two types of information for the purposes of simulation: information about collective benefits, and information about individual benefits. Based on the results from the previous study by Ramachandran et al. [21] it is assumed here that these two types of information have the most significant impact in relation to support for tidal energy. Information about collective benefits and information about individual benefits will henceforth be referred to as c-info and i-info, respectively. While input values for age and education can be taken from the statistical model on a scale of one to six, the values for c-info and i-info are represented through harvesting, receiving, and sharing rates in the ABS. While the individual outcome favourability and collective outcome favourability in the statistical model are based on a scale of zero to two, the c-info and i-info harvesting, receiving, and sharing rates in the ABS are on a scale of 0–1, with lower values in multinomial probit model corresponding to a lower values ABS and similarly, higher values in multinomial probit model corresponding to higher values in ABS.

In the ABS, the difference in the hierarchies based on their information preferences are manifest by the information harvesting and information receiving functions. From observations in the field, it is evident that the agents from top hierarchies have higher capability of harvesting information due to their greater access to information, as well as higher education [21]. Likewise, better education also allows agents to receive more information due to higher ability to process more information.

Based on field observation, this model assumes that information flow initiates from the top hierarchical group. Hence, in the initiation of the model, a single level 1 hierarchy member is created in the middle of the simulation environment with 100% of i-info and c-info while all other agents have no information. This model also assumes the location of the agent to be not highly significant, and hence all agents are located randomly such that no two agents are residing in the same cell. Cells are defined such that the centre cell has 100% of i-info and c-info and all other cells have no information. As the information flows through the environment, and between agents, the simulation calculates the number of information flow cycles it takes for the information to get to the probable agents who support tidal energy.

#### Model verification and validation

Before a simulation can be executed, it is important to verify and validate the model [50]. Schlesinger [51] defines verification and validation for the complicated system simulation in the field of operations research [51, 52]. In general, while verification of the model is defined substantiation that a computerized model represents a conceptual model within specified limits of accuracy, validation of the model is defined substantiation that a computerized model within its domain of applicability possesses a satisfactory range of accuracy consistent with the intended application of the model.

In the phase of verification, debugging which is to inspect the program to detect errors is implemented [53]. In this study, it is implemented by confirming that the simulation model is correctly coded, and the program performs as anticipated from the perspective of findings in the field. One of the key methods employed here for verification is *tracing—*a form of testing that comprises receiving all intermediate outputs from a computer program automatically, and then comparing it with manually calculated results [54]. To verify that the sharing and harvesting behaviours were working as anticipated, the attribute data for singular patches and agents were inspected, cycle-by-cycle, as the simulation proceeded under specially created test scenarios for which the correct behaviour was already known, and outputs were calculated manually. For these simulation experiments, the intermediate simulation outputs matched the manual calculations, thus supporting the verification of the model.

In the phase of validation, it must be confirmed that the behaviour of the created model and outputs from the model replicate a phenomenon in its actual state. Before any simulation model can be used to replicate a phenomenon in a society, it is important to validate the model for that community to ensure that the model is an accurate representation of the real-world system under study [53]. For this ABS model, primary data on the input parameters was collected using questionnaire survey, expert interviews, and field observations to identify the behaviours exhibited by different groups of individuals in the community [21]. Additionally, secondary sources such as the census data [55], is also employed to define the input parameters for the simulation. Moreover, regression analyses and other statistical measures as described earlier are employed to ensure the viability of the input parameters to replicate the actual situation of the case study area, as well as their use in the ABS. The validation of the simulation model can be accomplished by comparing the simulation outputs with the actual observations from the community.

### ABS model simulations

#### ABS base model

The general design of the model is inspired from the interactions between the members of the local community, and it replicates the ideal characteristics of the actual situation observed in Flores Timur Regency, including the hierarchical nature, age, education, and information preferences [21]. The simulation is arbitrarily set up with 2500 agents classified into four hierarchical groups reflecting the nature of society, with the number of agents in each group distributed according to the values shown in Table 3.

From the interviews with academics, as well as local community leaders, it was clear the society is structured hierarchically with varying influence for each group. Moreover, the education level also changed with the hierarchies, whereby the individuals belonging to the higher hierarchical groups tended to be more educated compared to others. Additionally, the more experienced stakeholders belonged to the higher hierarchical groups. This is reflected in the input parameters where values for age are set at 6, 5, 3, and 3 for groups 1, 2, 3, and 4, respectively, and values for education are set at 6, 4, 3, and 2 for groups 1, 2, 3, and 4, respectively. Based on further interviews and field observations, additional information was collected qualitatively to reflect the local situation in Flores Timur Regency, and is used to set up the remaining parameters for ABS. The harvesting, receiving, and sharing rates for i-info are set 0.05 more than c-info. The harvesting rates for groups 1, 2, 3, and 4, are set at 0.65, 0.65, 0.45, and 0.25, respectively, for c-info and 0.7, 0.7, 0.5, and 0.3, respectively, for i-info. The receiving rates for groups 1, 2, 3, and 4, are set at 0.75, 0.75, 0.55, and 0.35, respectively, for c-info and 0.8, 0.8, 0.6, and 0.4, respectively, for i-info. The sharing rate is set at 0.7 for c-info and 0.75 for i-info. The influence of each group also differs by 0.1 with values set at 1.0, 0.9, 0.8, and 0.7 for groups 1, 2, 3, and 4. Finally, the information-spread rate between cells is fixed at 0.5. This study assumes the above parameters and the assigned values reflect the situation in Flores Timur Regency accurately. Description of all the parameters used and their given values can be found in Additional file 1: Appendix S1.

The principle idea behind the base model is to replicate the flow of information as observed in a community like Flores Timur, where the information originates from the top level and spreads hierarchically, with the top level acquiring the information at the fastest rate compared to other groups. Additionally, the results of the simulation which enable us to identify strategies can help the project developers in implementing their information provision strategies by testing the impact of making additional information available from the environment, the influence of delayed involvement of agents from some hierarchical levels, and the effect of change in the number of agents in certain hierarchical levels.

GAMA Platform V1.8.0 is used for the simulation.

#### Additional simulations for parameter study

To assess the influence of information available from the environment, the information spread rates are set at varied amounts to simulate the spread of information through the environment. The simulation is performed two additional times with information spread rates set at 0.6 and 0.7, compared to value of 0.5 set for the base model.

To check if there is any significant effect of introducing certain hierarchical groups at varied times in the information flow process, two simulations are performed where level 3 and level 4 agents are introduced after 50 cycles and 70 cycles. Furthermore, two more simulations are executed where only the lowest level 4 agents are introduced in the information flow process after 50 cycles and 70 cycles. All other parameters were kept unaltered compared to the base model.

The impact of the number of agents in any hierarchical group was investigated by performing two additional simulations after altering the number of agents by adding 10% additional agents and 20% additional agents to level 2 hierarchy after removing the same number of agents from the lowest level 4. These simulations were repeated again with 10% additional agents and 20% additional agents in level 3, and reducing identical amount of agents from level 4. No other parameters apart from the agent numbers are altered in comparison with the base model.

## Results

### Post-estimation measures

The goodness-of-fit measures are presented in Table 4.

**Table 4:** Goodness-of-fit measures

Log-likelihood	− 125.260
LR (log-likelihood ratio)	250.521***
*R*-square	
Count	0.541
Count (adjusted)	0.184

*** Indicates *p*-value < 0.01

As shown in Table 4, the *R*-squared value is in the acceptable range pointing to a good fit of this model.

**Fig. 4:**
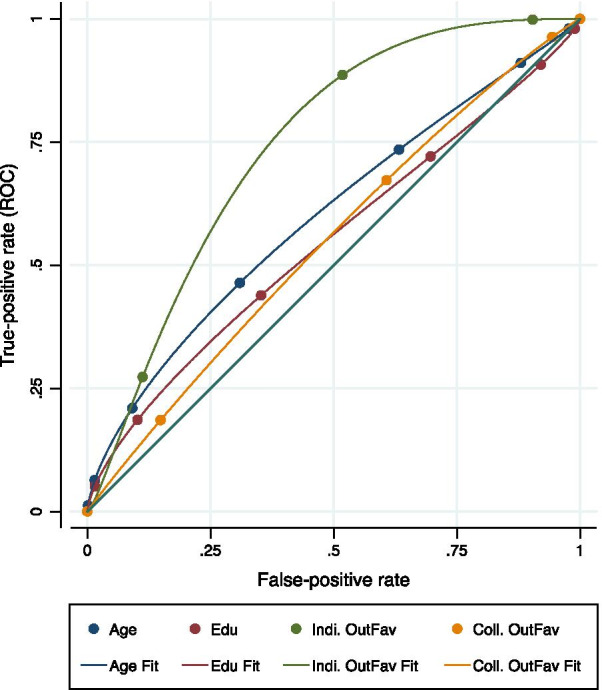
Receiver operating characteristic (ROC) curve

The ROC curve for the model is shown in Fig. 4.

As Fig. 4 shows, the curves lie above the diagonal line pointing to the higher accuracy of the variables employed.

Table 5 shows the area under the curve for the variables in this model.

**Table 5:** Area under the ROC curve

Variable	Obs	ROC area	Std. Err	95% conf. interval	
				5%	95%
Age	135	0.60	0.07	0.48	0.73
Edu	135	0.54	0.07	0.42	0.67
Indi. OutFav	135	0.72	0.05	0.62	0.81
Coll. OutFav	135	0.55	0.06	0.43	0.67

Coll. OutFav indicates the variable “Collective outcome favourability” asked in the field survey. It has three values 0, 1, and 2, respectively, denoting disagree, neither agree nor disagree, and agree

Indi. OutFav indicates the variable “Individual outcome favourability” asked in the field survey. It has three values 0, 1, and 2, respectively, denoting disagree, neither agree nor disagree, and agree

Education, asked in the field survey, is variable with values ranging from 1 to 6 denoting different categories

Age, asked in the field survey, is variable with values ranging from 1 to 6 denoting different categories

As described in Table 5, all the variables show ROC area values more than 0.5 pointing to the aptness of the variables.

After ensuring the viability of the four variables, the predicted probability to support tidal energy is estimated based on specific values for the variables. Table 6 shows the values for the four variables and the predicted probability of each hierarchical group to support tidal energy based on those values for the four variables, under all the three cases.

**Table 6:** Predicted probability of support for tidal energy at specified values for variables using multinomial probit

Hierarchy	Coll. OutFav	Indi. OutFav	Education	Age	Prob	
Case 1						
Level 1	0	2	6	6	0.65 (3.36)	***
Level 2	0	2	4	5	0.58 (3.56)	***
Level 3	0	2	3	3	0.38 (2.33)	**
Level 4	0	2	2	3	0.32 (1.89)	**
Case 2						
Level 1	0	2	6	6	0.65 (3.36)	***
Level 2	0	2	4	5	0.58 (3.56)	***
Level 3	1	2	3	3	0.30 (3.43)	***
Level 4	1	2	2	3	0.26 (2.51)	**
Case 3						
Level 1	1	2	6	6	0.57 (3.29)	***
Level 2	1	2	4	5	0.50 (4.57)	***
Level 3	0	2	3	3	0.38 (2.33)	**
Level 4	0	2	2	3	0.32 (1.89)	**

*z*-value is indicated in parentheses with probability

“Level” indicates social hierarchy. For instance, Level 1 denotes the top hierarchical group with level 4 denoting the bottom hierarchical group

Coll. OutFav indicates the variable “Collective outcome favourability” asked in the field survey. It has three values 0, 1, and 2, respectively, denoting disagree, neither agree nor disagree, and agree

Indi. OutFav indicates the variable “Individual outcome favourability” asked in the field survey. It has three values 0, 1, and 2, respectively, denoting disagree, neither agree nor disagree, and agree

Education, asked in the field survey, is variable with values ranging from 1 to 6 denoting different categories

Age, asked in the field survey, is variable with values ranging from 1 to 6 denoting different categories

*** and ** indicate *p*-value < 0.01 and 0.05, respectively

According to the multinomial probit estimations, the people belonging to the top hierarchical group show a probability of 57% or 65% to support tidal energy depending on the case. People belonging to the second group show a probability of 50% or 58% in support of tidal energy, and the third group shows a probability of 30% or 38%. Finally, 26% or 32% of people from group four will support tidal energy. Table 6 also shows that all the predicted probabilities to support tidal energy are statistically significant.

### The base model

**Fig. 5:**
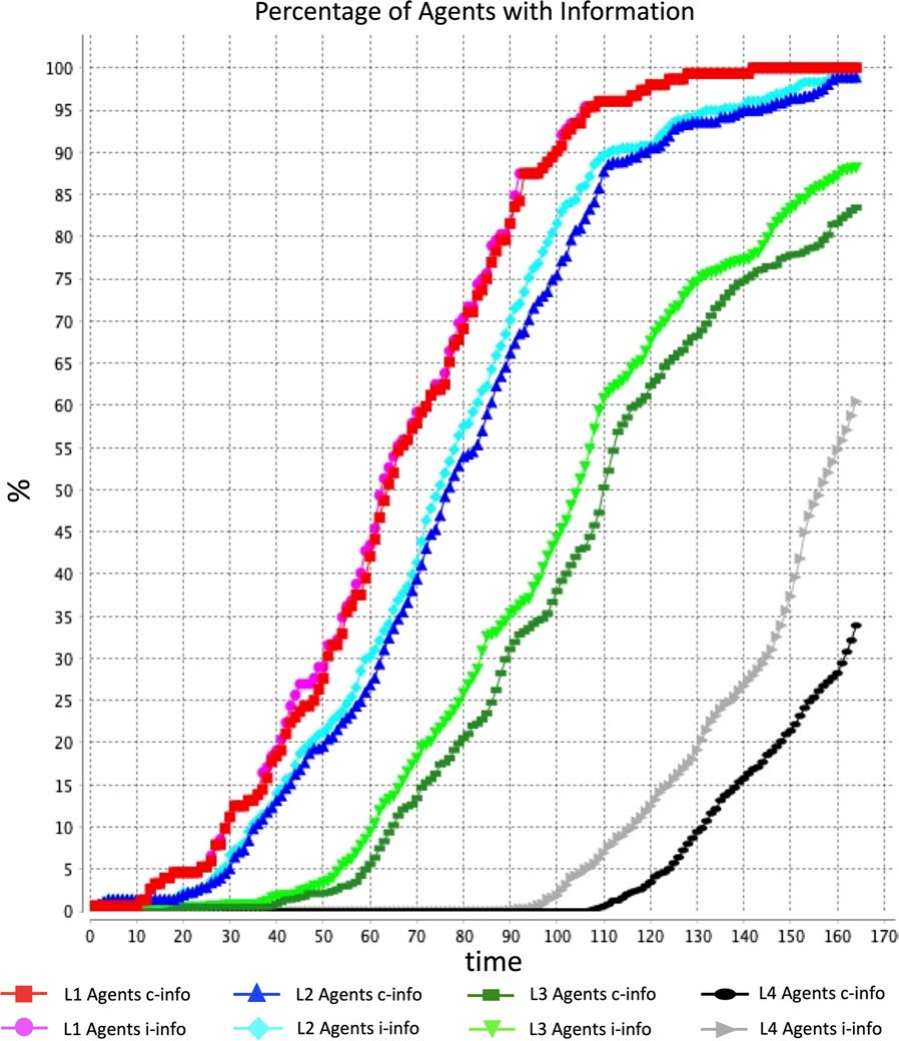
Graph showing the growth of information based on the agent hierarchical level and type of information (see Additional file 1: Appendix S1 for information about parameters)

Using the set parameters mentioned earlier for the base model simulation, the resulting graph showing the information growth among various hierarchical agent levels based on information type is shown in Fig. 5.

Results displaying the number of cycles it takes i-info and c-info to flow to the probable number of supporters of tidal energy are shown in Table 7.

**Table 7:** Number of cycles to disseminate information to probable supporters of tidal energy

Hierarchy	Prob	Info type	Number of cycles
Case 1			
Level 1	0.65	i-info	76
		c-info	76
Level 2	0.58	i-info	81
		c-info	84
Level 3	0.38	i-info	94
		c-info	99
Level 4	0.32	i-info	146
		c-info	162
Case 2			
Level 1	0.65	i-info	76
		c-info	76
Level 2	0.58	i-info	81
		c-info	84
Level 3	0.30	i-info	83
		c-info	89
Level 4	0.26	i-info	138
		c-info	155
Case 3			
Level 1	0.57	i-info	68
		c-info	68
Level 2	0.50	i-info	74
		c-info	76
Level 3	0.38	i-info	94
		c-info	99
Level 4	0.32	i-info	146
		c-info	162

i-info denotes information about individual benefits of tidal energy

c-info denotes information about collective benefits of tidal energy

Final column indicates the no. of cycles it takes for information to reach the probable number of supporters with the probability indicated in second column

Reflecting the hierarchical nature of the society, the agents from the top hierarchy, i.e. level 1 agents are the fastest to obtain information and agents from the bottom level, level 4 agents are the slowest to acquire information. Under case 1 and case 2, it takes 76 cycles to spread both types of information to probable supporters of tidal energy from top level 1 hierarchical group, however it takes 68 cycles under case 3. For group 2 agents under case 1 and case 2, it takes 81 cycles to spread i-info and 84 cycles to spread c-info among the probable supporters, while it takes 74 and 76 cycles to spread i-info and c-info, respectively, under case 3. Group 3 supporters who may support tidal energy take 94, 83, and 94 cycles to obtain i-info under cases 1, 2, and 3, respectively, and 99, 89, and 99 cycles to absorb c-info under respective cases 1, 2, and 3. Level 4 agents require 146 and 162 cycles for i-info and c-info flows under case 1, 138 and 155 cycles for i-info and c-info flows under case 2, and 146 and 162 cycles for i-info and c-info flows under case 3 to achieve the probable support for tidal energy.

Table 7 also shows case 3 is the fastest scenario for information flow to agents belonging to top hierarchical groups 1 and 2, and case 2 is the fastest scenario for agents belonging to the bottom hierarchical groups 3 and 4. Most importantly, case 1 can be characterized as the standard scenario for information flow among all hierarchical groups.

### Influence of information spread rate

**Fig. 6:**
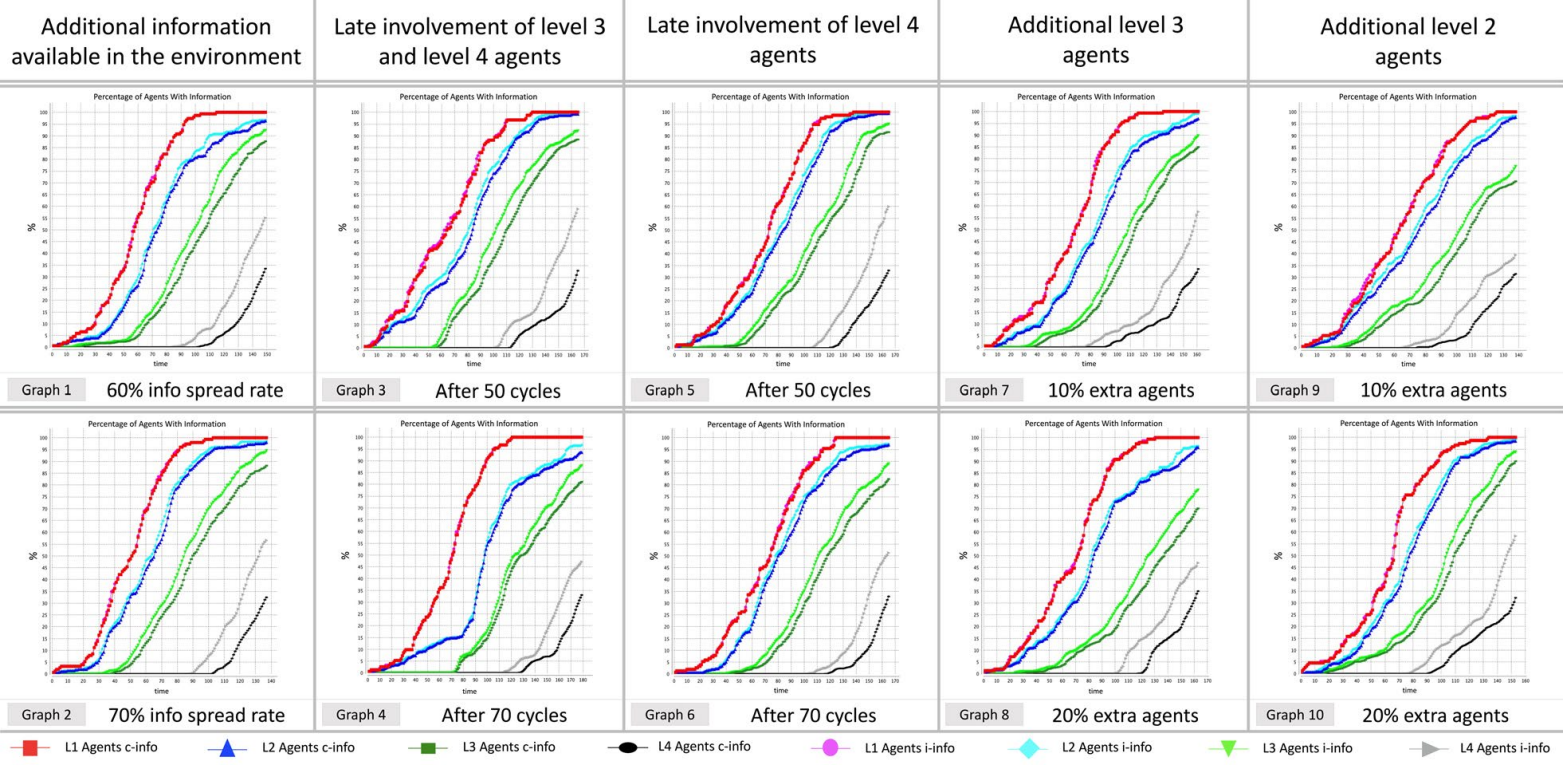
Graphs of various simulation approaches showing i-info and c-info acquired for each hierarchical group (see Additional file 1: Appendix S1 for information about parameters)

To identify the best pathways for information provision, certain parameters are altered compared to the base model. Results from all approaches to the simulation, showing the information growth of c-info and i-info for all agents are summarized in Fig. 6.

Table 8 shows the number of cycles it takes for information to flow to probable supporters of tidal energy in each hierarchical group under varied simulation conditions.

**Table 8:** Number of cycles to disseminate information to probable supporters of tidal energy under various approaches

Hierarchy	Prob	Info type	Number of cycles										
			Base model	Graph 1	Graph 2	Graph 3	Graph 4	Graph 5	Graph 6	Graph 7	Graph 8	Graph 9	Graph 10
Case 1													
Level 1	0.65	i-info	76	64	57	76	77	83	82	79	77	73	68
		c-info	76	65	57	76	78	84	83	79	78	73	69
Level 2	0.58	i-info	81	76	67	88	101	88	86	88	87	75	74
		c-info	84	78	70	90	103	90	88	91	90	78	76
Level 3	0.38	i-info	94	88	77	109	112	97	101	97	109	95	99
		c-info	99	94	83	115	115	103	107	104	118	100	104
Level 4	0.32	i-info	146	132	121	148	162	145	149	142	146	121	137
		c-info	162	148	136	164	178	164	164	160	162	137	152
Case 2													
Level 1	0.65	i-info	76	64	57	76	77	83	82	79	77	73	68
		c-info	76	65	57	76	78	84	83	79	78	73	69
Level 2	0.58	i-info	81	76	67	88	101	88	86	88	87	75	74
		c-info	84	78	70	90	103	90	88	91	90	78	76
Level 3	0.30	i-info	83	82	71	103	109	92	95	92	101	84	94
		c-info	89	89	77	107	112	97	100	98	108	92	98
Level 4	0.26	i-info	138	128	118	144	157	140	145	134	141	114	131
		c-info	155	143	131	161	173	158	159	151	157	128	148
Case 3													
Level 1	0.57	i-info	68	61	54	72	73	76	77	73	75	68	66
		c-info	68	61	54	72	73	76	77	74	75	68	66
Level 2	0.50	i-info	74	70	63	84	97	80	78	83	82	70	70
		c-info	76	73	65	85	97	83	80	85	83	72	72
Level 3	0.38	i-info	94	88	77	109	112	97	101	97	109	95	99
		c-info	99	94	83	115	115	103	107	104	118	100	104
Level 4	0.32	i-info	146	132	121	148	162	145	149	142	146	121	137
		c-info	162	148	136	164	178	164	164	160	162	137	152

i-info denotes information about individual benefits of tidal energy

c-info denotes information about collective benefits of tidal energy

Graphs 1–10 indicate the various simulation approaches presented through graphs 1–10 in Fig. 6

When the amount of information available in the environment is altered by increasing the information spread rate, the amount of information acquired by the agents changes significantly. Graphs 1 and 2 in Fig. 6 show the amount of information possessed by various agent groups when information spread rate in the environment is at 60% and 70%, respectively, compared to the base model where the information spread rate is at 50%. Table 8 shows that information flow to the probable supporters of tidal energy is much faster when there is more information available in the environment for all the hierarchical groups in all the three cases.

In the instance of graph 1, characterized by 60% information spread rate, i-info takes on average 8 cycles less than the base model to spread to the probable supporters of tidal energy in all the hierarchical groups for the first case. Similarly, i-info, on average, takes six cycles less for the second case and eight cycles less for the third case to spread to all groups. Likewise, c-info takes on average 8, 7, and 7 cycles less than the base model for case 1, case 2, and case 3, respectively.

When the information spread rate is at 70% as exemplified in graph 2, information flow process becomes significantly faster. It takes i-info, on average, 19, 16, and 17 fewer cycles than the base model for case 1, case 2, and case 3, respectively, to flow to the probable supporters of tidal energy in all hierarchical groups. Likewise, c-info takes 19, 17, and 17 cycles less than the base model on average for case 1, case 2, and case 3, respectively.

### Later involvement of lower hierarchical level agents

Graphs 3 and 4 in Fig. 6 show the information growth when agents from lower hierarchical levels, groups 3 and 4, get involved in the information flow process only after 50 cycles and 70 cycles, respectively. According to the results shown in Table 8, the information flows 6–8 cycles slower for graph 3 and 13–16 cycles slower for graph 4 compared to the base model, on average for all the groups under all the case parameters.

Graphs 5 and 6 in Fig. 6 show the information growth when agents from the bottom hierarchical level group 4, get involved in the information flow process only after 50 cycles and 70 cycles, respectively. Table 8 shows that the information flows 4–6 cycles slower for graph 5 and 5–7 cycles slower for graph 6 compared to the base model, on average for all the groups under all the case parameters.

### Different level-wise distribution of agents

Graphs 7 and 8 in Fig. 6 show the information growth when the number of agents is increased in level 3 with the equal number of agents reduced in level 4. When the proportion of agents from level 3 is increased by 10%, the information flow takes 2–4 cycles compared to the base model longer to get to all the agent groups under all the cases as shown by graph 7 in Table 8. Similarly, when there are 20% additional level 3 agents, the information flow is prolonged by a further 5–8 cycles for all the hierarchical groups as shown by graph 8 in Table 8.

Graphs 9 and 10 in Fig. 6 show the information growth when the number of agents is increased in level 2 with the equal number of agents reduced in level 4. When the proportion of agents from level 2 is increased by 10%, the information flow becomes faster by 7–8 cycles compared to the base model as shown by graph 9 in Table 8. Similarly, when there are 20% additional level 2 agents, the information flow is shortened by 2–5 cycles for all the hierarchical groups as shown by graph 10 in Table 8.

## Discussion

### Efficient information dissemination

In areas such as diffusion of an innovation, or spread of panic or riots, transmission of diseases, or public health advisories, the study of how information is generated, and the dynamics affecting how it is disseminated, has a prominent position in the field of social sciences. However, few studies have explored information dissemination strategies for increasing support of renewable energy technologies. In this context, this study highlights vital strategies that can lead to increase in support for tidal energy by identifying the key factors that affect the flow of information in a local community.

Information dissemination activities can be more efficient when the type of information that is more effective locally is diffused in the community. As the results from the post-estimation ROC curve indicate, individual outcome favourability is the most significant variable in driving support for tidal energy. Accordingly, as ABS shows i-info spreads faster through all hierarchical groups compared to c-info, the developers should focus more on disseminating information that focuses on benefits of tidal energy that specifically cater to the individual, as opposed to the information that concentrates on community benefits. For example, the developers should focus more on disseminating information related to additional jobs that benefit individuals, compared to the information about infrastructure development, which supports the whole community.

#### Short-term strategies

**Fig. 7:**
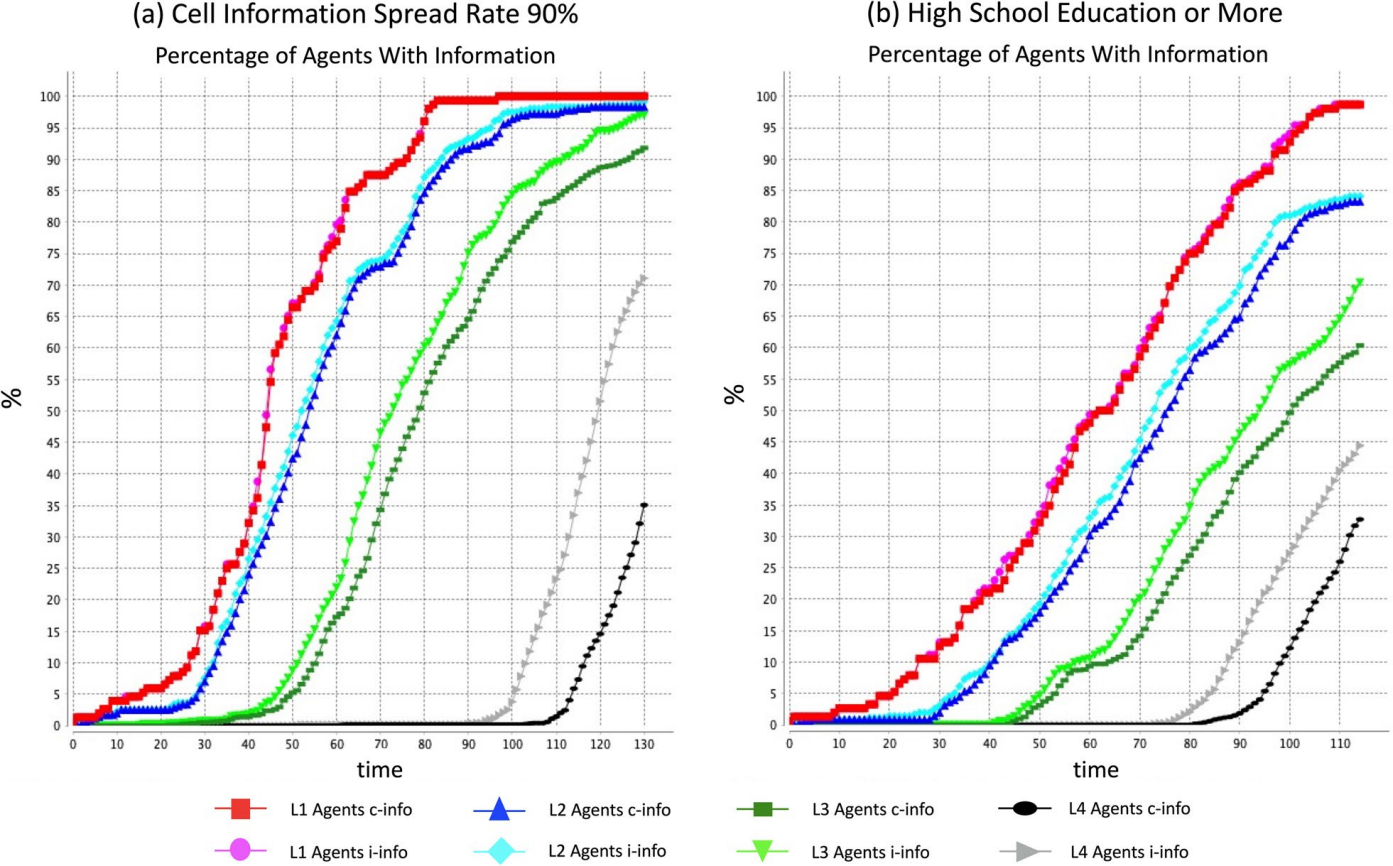
Information growth for all hierarchical groups with **a** 90% cell information spread rate and **b** at least high school education for all agents

When the information available from the environment is very low, the agent information levels are similarly at low level. However, when there is additional information is available to be absorbed by the agents, even the agents belonging to low hierarchy are able to gain information. Figure 7a shows the information growth for all hierarchical groups when the cell-to-cell information spread rate is at 90% resulting in abundance of information available in the environment. Table 9a shows the number of cycles needed to spread information to the probable supporters of tidal energy. With additional information available, information takes 25–29 cycles less on average compared to the base model to be absorbed by probable supporters of tidal energy.

**Table 9:** Number of cycles to spread information to probable supporters of tidal energy with (a) high information availability due to 90% information spread rate and (b) higher education values when every agent has at least high school education

Hierarchy	Prob	Info type	Number of cycles		
			Base model	(a) 90% cell info spread rate	(b) High school education or more
Case 1					
Level 1	0.65	i-info	76	48	73
		c-info	76	49	74
Level 2	0.58	i-info	81	56	78
		c-info	84	57	80
Level 3	0.38	i-info	94	66	81
		c-info	99	71	88
Level 4	0.32	i-info	146	112	103
		c-info	162	129	113
Case 2					
Level 1	0.65	i-info	76	48	73
		c-info	76	49	74
Level 2	0.58	i-info	81	56	78
		c-info	84	57	80
Level 3	0.30	i-info	83	63	77
		c-info	89	68	83
Level 4	0.26	i-info	138	111	98
		c-info	155	126	109
Case 3					
Level 1	0.57	i-info	68	45	68
		c-info	68	45	69
Level 2	0.50	i-info	74	52	72
		c-info	76	53	75
Level 3	0.38	i-info	94	66	81
		c-info	99	71	88
Level 4	0.32	i-info	146	112	103
		c-info	162	129	113

i-info denotes information about individual benefits of tidal energy

c-info denotes information about collective benefits of tidal energy

The developers should focus on getting as much information as possible available to the public by not only relying on mouth-to-mouth communication, but also by employing traditional promotional methods such as newspaper and television advertisements, or employing more modern approaches such as promotions through social media applications that are becoming increasingly popular in Flores Timur. Additionally, during discussions with the local government officers as well as members of the general public, it emerged that the locals currently the locals use the television most frequently for acquiring information, and so the developers should consider using local television as a method to provide additional information to the public to achieve maximum impact.

Developers should also take full advantage of current local practices of information delivery through community consultation workshops known as ‘socialization’ events in Flores Timur Regency. Developers can strategize on how they conduct their socialization sessions or who to focus their advertisements on. The results also show that there is low impact on the information flow when the lower hierarchy agents are introduced in the information flow only at later stages. In practice, the developers can design their promotional activities separately for the higher hierarchical groups, who can be targeted earlier, compared to the lower hierarchical groups who make up the bulk of the population and can be included later. For instance, the developers can hold the socialization sessions with only the most influential local stakeholders in the initial stages, thereby make savings in terms of cost, time, and effort that may otherwise be spent on mass promotional activities.

This strategy is not only significant to realize the savings, but also extremely important if one considers the current practices in Indonesia where the local population looks to the influential figures known as *‘tokoh masyarakat’*, such as prominent politicians or religious leaders, for advice on all matters of public importance. In recent years, *‘tokoh masyarakat’* have been effective in informing the public on the ways to fight against the Covid-19 pandemic using their knowledge and attitudes [56]. In a similar manner, *‘tokoh masyarakat’* can be provided with complex information such as scientific data from environmental agencies or fishery departments highlighting the benefits to the farmers and fishermen. They can then use their knowledge, influence, and leadership to disseminate the information in a simplified way that everyone can understand, and hence educate the society as well as increase the support for tidal energy.

#### Long-term strategies

Additionally, the developers can also focus on long-term plans that go beyond increasing information provision for a few cycles. The effectiveness of the information flow is highest when the community is well educated, and adds on to the point made by the head of the non-formal education in Flores Timur who emphasized the role of education in the development of Flores Timur Regency. In Flores Timur only 11.38% of the population have a high school diploma, with only 3.31% having college level education. Strikingly however, 32.15% of those aged over 10 years, have not graduated from any institution providing formal education [55]. Figure 7b shows the information growth for all hierarchical groups when all the agents possess high school education or more. Table 9b shows the number of cycles to spread information efficiently to the probable supporters of tidal energy when all the agents have at least high school level education. As displayed in Table 9b, information takes 13–16 cycles less on average to be absorbed by probable supporters of tidal energy. While this is an ideal scenario and may take many years to implement successfully, it shows excellent results in making information flow faster and more efficient, especially for the masses belonging to the lowest hierarchical group 4 who take 40–49 cycles less on average under all the three cases.

While the government and policy makers focus on improving the current infrastructure for formal school education, the developers can also get further involved in the community by focusing their CSR efforts on community educational activities. Project developers can team up with NGOs and community organizations to promote adult education programmes in the society as has been practised in other areas, thereby directly contributing to increasing literacy rate of the local society, while indirectly benefitting with the higher levels of support for their project [57–60]. This observation further validates the opinion made in an interview by the head of the non-formal education in Flores Timur who emphasized the role of education in the overall development of Flores Timur Regency. In this way the project developers not only directly contribute on attaining SDG 7, but also make efforts toward realizing SDG 4: Education [61].

## Conclusions and limitations

### Conclusion

The purpose of this study is to identify the most efficient strategies for information provision to promote tidal energy in a remote isolated area of a developing country through simulation of agent-based simulation (ABS) model built with the estimation results of Multinomial probit model which is extended from Ramachandran et al. [21]. The notable feature of this paper is combining the simulation by the scenario from field observation with validated estimation results of multinominal probit model for questionnaire survey to reflect the actual status of case area, which is Flores Timur Regency, Indonesia. After validating the results of multinominal probit model by post-estimation, factors for the ABS are defined from field observation: social hierarchy, and information types which are i-info (information about individual benefits) and c-info (information about collective benefits).

The ability of the created model to capture real-world pattern of information flow in Flores Timur Regency indicates that the findings may have significant insights contributing to successful deployment of tidal energy in Indonesia. Information pertaining to the individual benefits of tidal energy deployment in Flores Timur is most effective when pursuing public engagement strategies based on information provision as proven by results from the multinomial probit modelling. Information provision strategies are highly effective when there is additional information available to be absorbed by the local population as graphs 1 and 2 in Table 8 show that information flows fastest to all hierarchical groups when the information-spreading rate is increased.

Additionally, specific implications can also be derived from the results of the study. The developers can strategize information provision by focusing on the most influential stakeholders initially, before including all other locals, thereby saving cost, time, and effort—a strategy based on results displayed in graphs 3 to 6 in Table 8 which show the minimal impact of late involvement of certain hierarchical groups in information flow process. Finally, the most effective strategy, though long-term, involves having highly educated local population, which can aid the developers in making inroads with the local community by engaging in education programmes resulting in multiple mutual benefits.

This study based on agent-based simulation not only addresses the lack of studies for tidal energy in developing countries, but it does so from the perspective of project developers, while proposing pathways for information provision strategies necessary to sustainably deploy tidal energy projects. Additionally, the short, medium, and long-term strategies outlined in this study contribute toward achievement of multiple SDGs.

### Limitations

Expectedly, the model is a simplification of the real-world system, with several improvements that can be considered for future research. First, this simulation focuses on mouth-to-mouth information flow between the agents and the impact of increasing availability of communication technologies has not been captured. Arguably, the use of modern social media communication applications may have a significant impact on the flow of information, which is not specifically addressed in the model. Second, this model assumes the initial information level to be equal for all the agents, while in practical terms, the initial information about tidal energy may differ from person to person. Third, this study only considers two types of information—c-info and i-info—that can increase support for tidal energy as revealed by Ramachandran et al. [21], however, in some cases, new information such as impact on marine life or the visible infrastructure, can potentially reduce support for marine energy [19]. Fourth, this model assigns the location in the environment on a random basis, though it may be possible that agents belonging to various hierarchical groups might be found together in clusters. Finally, this model assumes a constant rate of information dissemination through all the cycles. Studies show the rate of information diffusion to be high when the process of dissemination begins, but it gradually tends to dwindle down to lower rates. Moreover, as information disseminates from person to person, the natural trend is an erosion of the initial information [62].

## Supplementary Information

**Additional file 1: Appendix S1. **Simulation input parameters.

## Data Availability

All data generated or analysed during this study are available from the corresponding author on reasonable request.

## Abbreviations

SDG: Sustainable Development Goal
ABS: Agent-based simulation
ROC: Receiving operating characteristic
