# Supplementary material for: Information flow to increase support for tidal energy development in remote islands of a developing country: agent-based simulation of information flow in Flores Timur Regency, Indonesia
Source: Energy Sustain Soc. 2021 Jul 21;11(1):26. doi: 10.1186/s13705-021-00302-8 (PMC8294289; doi:10.1186/s13705-021-00302-8)
Supplement: Supplementary file 1 — Additional file 1: Appendix S1. Simulation input parameters. [file 13705_2021_302_MOESM1_ESM.pdf]

Appendix 1

Simulation Input Parameters

Input Parameters	Base Model	Graph 1	Graph 2	Graph 3	Graph 4	Graph 5	Graph 6	Graph 7	Graph 8	Graph 9	Graph 10
**No. of agents**											
Level 1	152	152	152	152	152	152	152	152	152	152	152
Level 2	358	358	358	358	358	358	358	358	358	394	430
Level 3	804	804	804	804	804	804	804	884	964	804	804
Level 4	1186	1186	1186	1186	1186	1186	1186	1106	1026	1150	1114
**Age**											
Level 1	6.00	6.00	6.00	6.00	6.00	6.00	6.00	6.00	6.00	6.00	6.00
Level 2	5.00	5.00	5.00	5.00	5.00	5.00	5.00	5.00	5.00	5.00	5.00
Level 3	3.00	3.00	3.00	3.00	3.00	3.00	3.00	3.00	3.00	3.00	3.00
Level 4	3.00	3.00	3.00	3.00	3.00	3.00	3.00	3.00	3.00	3.00	3.00
**Education**											
Level 1	6.00	6.00	6.00	6.00	6.00	6.00	6.00	6.00	6.00	6.00	6.00
Level 2	4.00	4.00	4.00	4.00	4.00	4.00	4.00	4.00	4.00	4.00	4.00
Level 3	3.00	3.00	3.00	3.00	3.00	3.00	3.00	3.00	3.00	3.00	3.00
Level 4	2.00	2.00	2.00	2.00	2.00	2.00	2.00	2.00	2.00	2.00	2.00
**Influence**											
Level 1	1.00	1.00	1.00	1.00	1.00	1.00	1.00	1.00	1.00	1.00	1.00
Level 2	0.90	0.90	0.90	0.90	0.90	0.90	0.90	0.90	0.90	0.90	0.90
Level 3	0.80	0.80	0.80	0.80	0.80	0.80	0.80	0.80	0.80	0.80	0.80
Level 4	0.70	0.70	0.70	0.70	0.70	0.70	0.70	0.70	0.70	0.70	0.70
**Threshold**											
Agent c-info	0.10	0.10	0.10	0.10	0.10	0.10	0.10	0.10	0.10	0.10	0.10
Agent i-info	0.10	0.10	0.10	0.10	0.10	0.10	0.10	0.10	0.10	0.10	0.10
Cell c-info	0.10	0.10	0.10	0.10	0.10	0.10	0.10	0.10	0.10	0.10	0.10
Cell i-info	0.10	0.10	0.10	0.10	0.10	0.10	0.10	0.10	0.10	0.10	0.10
**Cell spreading rate**											
c-info	0.50	0.60	0.70	0.50	0.50	0.50	0.50	0.50	0.50	0.50	0.50
i-info	0.50	0.60	0.70	0.50	0.50	0.50	0.50	0.50	0.50	0.50	0.50
**Agent c-info harvesting rate**											
Level 1	0.65	0.65	0.65	0.65	0.65	0.65	0.65	0.65	0.65	0.65	0.65
Level 2	0.65	0.65	0.65	0.65	0.65	0.65	0.65	0.65	0.65	0.65	0.65
Level 3	0.45	0.45	0.45	0.45	0.45	0.45	0.45	0.45	0.45	0.45	0.45
Level 4	0.25	0.25	0.25	0.25	0.25	0.25	0.25	0.25	0.25	0.25	0.25
**Agent i-info harvesting rate**											
Level 1	0.70	0.70	0.70	0.70	0.70	0.70	0.70	0.70	0.70	0.70	0.70
Level 2	0.70	0.70	0.70	0.70	0.70	0.70	0.70	0.70	0.70	0.70	0.70
Level 3	0.50	0.50	0.50	0.50	0.50	0.50	0.50	0.50	0.50	0.50	0.50
Level 4	0.30	0.30	0.30	0.30	0.30	0.30	0.30	0.30	0.30	0.30	0.30
**Agent c-info receiving rate**											
Level 1	0.75	0.75	0.75	0.75	0.75	0.75	0.75	0.75	0.75	0.75	0.75
Level 2	0.75	0.75	0.75	0.75	0.75	0.75	0.75	0.75	0.75	0.75	0.75
Level 3	0.55	0.55	0.55	0.55	0.55	0.55	0.55	0.55	0.55	0.55	0.55
Level 4	0.35	0.35	0.35	0.35	0.35	0.35	0.35	0.35	0.35	0.35	0.35
**Agent i-info receiving rate**											
Level 1	0.80	0.80	0.80	0.80	0.80	0.80	0.80	0.80	0.80	0.80	0.80
Level 2	0.80	0.80	0.80	0.80	0.80	0.80	0.80	0.80	0.80	0.80	0.80
Level 3	0.60	0.60	0.60	0.60	0.60	0.60	0.60	0.60	0.60	0.60	0.60
Level 4	0.40	0.40	0.40	0.40	0.40	0.40	0.40	0.40	0.40	0.40	0.40
**Agent c-info sharing rate**											
Level 1	0.70	0.70	0.70	0.70	0.70	0.70	0.70	0.70	0.70	0.70	0.70
Level 2	0.70	0.70	0.70	0.70	0.70	0.70	0.70	0.70	0.70	0.70	0.70
Level 3	0.70	0.70	0.70	0.70	0.70	0.70	0.70	0.70	0.70	0.70	0.70
Level 4	0.70	0.70	0.70	0.70	0.70	0.70	0.70	0.70	0.70	0.70	0.70
**Agent i-info sharing rate**											
Level 1	0.75	0.75	0.75	0.75	0.75	0.75	0.75	0.75	0.75	0.75	0.75
Level 2	0.75	0.75	0.75	0.75	0.75	0.75	0.75	0.75	0.75	0.75	0.75
Level 3	0.75	0.75	0.75	0.75	0.75	0.75	0.75	0.75	0.75	0.75	0.75
Level 4	0.75	0.75	0.75	0.75	0.75	0.75	0.75	0.75	0.75	0.75	0.75
**Initial involvement cycle**											
Level 1	1	1	1	1	1	1	1	1	1	1	1
Level 2	1	1	1	1	1	1	1	1	1	1	1
Level 3	1	1	1	50	70	1	1	1	1	1	1
Level 4	1	1	1	50	70	50	70	1	1	1	1
**Visibility range**	0.50	0.50	0.50	0.50	0.50	0.50	0.50	0.50	0.50	0.50	0.50
